# Characterization of 15 Polymorphic Microsatellite Loci for *Cephalotaxus oliveri* (Cephalotaxaceae), a Conifer of Medicinal Importance

**DOI:** 10.3390/ijms130911165

**Published:** 2012-09-07

**Authors:** Yingchun Miao, Xuedong Lang, Shuaifeng Li, Jianrong Su, Yuehua Wang

**Affiliations:** 1 Department of Botany, School of Life Sciences, Yunnan University, Kunming 650091, China; E-Mail: myczsh@163.com; 2 Research Institute of Resource Insects, Chinese Academy of Forest (CAF), Kunming 650224, China; E-Mails: langxuedong@gmail.com (X.L.); shuaifengli@163.com (S.L.)

**Keywords:** *Cephalotaxus oliveri*, FIASCO, microsatellite loci, PCR amplification

## Abstract

*Cephalotaxus oliveri* is a scarce medicinal conifer endemic to the south central region of China and Vietnam. A small fragmented population presently exists due to anthropogenic disturbance. *C. oliveri* has been used for its alkaloids harringtonine and homoharringtonine, which are effective against leucocythemia and lymphadenosarcoma. Monoecious plants have been detected in nature, although they were understood to be dioecious. In order to study the mating system, population genetics and the genetic effects of habitat fragmentation on *C. oliveri*, 15 polymorphic and 12 monomorphic microsatellite loci were developed for *C. oliveri* by using the Fast Isolation by AFLP of Sequences Containing repeats (FIASCO) protocol. The polymorphisms were assessed in 96 individuals from three natural populations (32 individuals per population). The number of alleles per locus ranged from two to 33, the observed and expected heterozygosity per locus ranged from 0.000 to 1.000 and from 0.000 to 0.923, respectively. These loci would facilitate a comprehensive understanding of the genetic dynamics on *C. oliveri*, which will be useful for establishing effective conservation strategies for this species.

## 1. Introduction

*Cephalotaxus oliveri* Masters (Cephalotaxaceae) is a scarce medicinal conifer endemic to the south central region of China and Vietnam [1]. It is a Tertiary species of *Cephalotaxus* [2], and is well-known for producing the alkaloids harringtonine and homoharringtonine, which are effective against leucocythemia and lymphadenosarcoma [3]. Moreover, the seed oil is used for cooking, the wood for furniture, leaf extracts can be used for adhesives, and the tree itself is cultivated as an ornamental. However, due to limited natural reproduction, excessive exploitation and destruction of its natural habitats over past decades, a small fragmented population presently exists. The species has been placed under state protection and listed as vulnerable in the IUCN Red List (http://www.iucnredlist.org).

*C. oliveri* was understood to be dioecious [4]. However, we have recently detected monoecious plants in Zheneng Country (24°17′56.4″N/101°21′E), Xinping County, Yunnan Province. Thus, there is a need for a comprehensive analysis of the mating system, population genetics and genetic effects of habitat fragmentation. Unfortunately, few studies have occurred due to the lack of codominant markers, such as microsatellites. Based on their codominant and hypervariable nature, microsatellites have become ideal markers in mating system [5] and population genetics analysis [6]. Presently only 13 polymorphic and 6 monomorphic microsatellite loci have been developed in *C. oliveri*. [7]. However, Koskinen *et al.* [8] had suggested it was important to maximize the number of microsatellites to obtain correct phylograms and increase the stability of genetic distances and corresponding phylograms even if optimally high microsatellite numbers (>30) were out of reach when studying population genetics. Furthermore, motif types and motif lengths have a significant effect on microsatellite variability [9]. Thus, 15 polymorphic and 12 monomorphic microsatellite loci with other various motif types and motif lengths for *C. oliveri* are characterized here. The total 46 microsatellite loci developed by us and Pan *et al.* [7] would be useful for a comprehensive understanding of the mating system, population genetics and genetic effects of habitat fragmentation, and a basis for studying microsatellite evolution and dynamics [10], genetic mapping [11] and molecular breeding [12] of the species in the future.

## 2. Results and Discussion

A total of 1056 white positive clones were captured, among which only 312 (29.5%) microsatellite clones were detected. Seventy-nine (25.3%) microsatellite sequences with sufficient flanking regions were used to design primers. As a result, only 27 primers (34.2%) produced clear and reliable bands, and were tested polymorphism in 96 individuals from three natural populations (32 individuals per population). Fifteen of the 27 loci in *C. oliveri* were polymorphic (CO1–CO15), and 12 (CO16–CO27) were monomorphic (Table 1).

No significant linkage disequilibrium was detected after Bonferroni correction (*p* < 0.05). The number of alleles per locus ranged from 2 to 33, with a total of 206 different alleles scored in 96 individuals. The observed and expected heterozygosity varied from 0.000 to 1.000 and 0.092 to 0.923, respectively (Table 2). Most loci in the three populations did not significantly deviate from HWE (Table 2). Estimated frequencies of null alleles were very low for 14 loci, varying from 0%–9% with a mean of 3%, except the locus CO2 characterized with 24% (Table 2). CO2 significantly deviated from HWE after Bonferroni correction (*p* < 0.00111) for the three populations due to heterozygote deficiency, suggesting the possibility of existence of null alleles in this locus.

## 3. Experimental Section

Three microsatellite-enriched libraries were constructed following the FIASCO (Fast Isolation by AFLP of Sequences Containing repeats) protocol [13]. Genomic DNA of a single individual of *C. oliveri* was extracted from dried leaves following the cetyltrimethyl ammonium bromide (CTAB) method [14]. About 500 ng genome DNA was completely digested with *Mse*I (Fermentas, Glen Burmie, Maryland, USA) at 65 °C for 3 h, and then ligated to an *Mse*I adaptor pair (5′-TACTCAGGACTCAT-3′/5′-GACGATGAGTCCTGAG-3′) using T4 DNA ligase (Fermentas, Glen Burmie, Maryland, USA). Five microliters of ligated DNA fragments diluted 10-fold were amplified with adapter-specific primer *Mse*I-N (5′-GATGAGTCCTGAGTAAN-3′) in a 20 μL reaction mixture containing 2.0 mM MgCl_2_, 1.25 pM *Mse*I-N primer, 0.2 mM dNTP, 1 U *Taq* DNA polymerase (TransGen, Beijing, China). PCR amplification was as follows: 95 °C for 3 min, 23 cycles of 94 °C for 1 min, 53 °C for 1 min, 72 °C for 1 min, followed by 72 °C for 10 min. To enrich the fragments containing microsatellite repeats, 100 μL of PCR products were denatured at 95 °C for 5 min, then hybridized with four 1 μM 5′-biotinylated microsatellite probes [library 1 = (AG)_15_ and (AC)_15_; library 2 = (CT)_15_, and library 3 = (AAG)_10_] at 48 °C for 2 h, 55 °C for 3 h, and 52 °C for 2 h, respectively. DNA fragments containing targeted repeats were selectively captured by magnetic streptavidin-coated particles (Promega, Madison, Wisconsin, USA). Recovered microsatellite DNA fragments were amplified with *Mse*I-N by 30 cycles according to the conditions described above. About 20 ng PCR product with a range of 200 to 800 bp purified via the E.Z.N.A. Gel Extraction kit (Omega Bio-Tek, Winooski, Vermont, USA) were directly ligated into a pEASY-T1 Cloning vector (TransGen, Beijing, China), and then transformed into *Escherichia coli* DH5a competent cells (Biomed, Beijing, China). Positive clones were selected using blue/white screening after growing on IPTG-XGal media, and further tested by PCR with M13 primers (5′-GTAAAACGACGGCCAGT-3′/5′-CAGGAAACAGCTATGAC-3′) and the corresponding primer-mix (AG)_15_ and (AC)_15_, or (CT)_15_, and or (AAG)_10_, respectively, for confirming positive clones containing microsatellites. PCR amplification was performed in a 15 μL reaction mixture containing 2 μL of bacterial suspension, 2.0 mM MgCl_2_, 0.2 μM each primer, 0.2 mM dNTP, 1 U *Taq* DNA polymerase (TransGen, Beijing, China). PCR amplification was as follows: 95 °C for 3 min, 35 cycles of 94 °C for 30 s, 55 °C for 30 s, 72 °C for 30 s, followed by 72 °C for 10 min. Detected microsatellite clones were sequenced by an ABI3730xl Genetic Analyzer (Applied Biosystems, Carlsbad, California, USA). Microsatellite sequences with sufficient flanking regions were used to design primers with Primer 5 [15]. Ninety-six individuals in three natural populations (32 per population) from Daozhen County (DZ) (29°4′55.2″N, 107°24′54″E), Guizhou Province; Pingbian (PB) (24°59′56.4″N, 101°21′E) County, Yunnan Province; and Luxi (LX) (27°16′34.6″N, 114°4′33.5″E) County, Jiangxi Province were used for primer testing. Herbarium voucher (Daozhen: *L.X.D. 09-103*; Pingbian: *L.X.D. 09-275*; Luxi: *L.X.D. 10-366*) deposited in Yunnan University Herbarium Laboratory of Pteridophyta (PYU), Kunming, Yunnan, China. We labeled 6-FAM at the 5′ end of each forward primer for the loci showed clear and reliable bands.

The above 96 individuals were also used to test the polymorphism of these workable microsatellite primers. Microsatellite loci were amplified in 20 μL volume with approximately 25 ng genomic DNA as the template, 2.0 mM MgCl_2_, 0.5 mM each primer, 0.25 mM dNTP, 1.5 U *Taq* DNA polymerase (TransGen, Beijing, China). PCR amplification was as follows: 95 °C for 3 min; 35 cycles of 94 °C for 30 s, annealing at optimal temperature 49 °C–66 °C for 30 s (see Table 1), and 72 °C for 30 s, followed by 72 °C for 10 min. These microsatellite fragments were separated on an ABI 3730xl genetic analyzer (Applied Biosystems, Carlsbad, CA, USA) with GenScan-500 size standard. All alleles were scored with the help of GeneMapper 4.

The linkage disequilibrium between loci and number of alleles per locus (*N*_a_) per population were estimated using FSTAT 2.9.3 (A program to estimate and test gene diversities and fixation indices) [16]. The observed (*H*_o_) and expected (*H*_e_) heterozygosities were calculated by ARLEQUIN 3.01[17]. The significant test of Hardy-Weinberg equilibrium (HWE) was done in GENEPOP 4.0.9 [18]. Lastly, the frequencies of null alleles per locus were calculated with FreeNA [19].

## 4. Conclusions

In summary, 15 polymorphic microsatellite markers have been specifically developed for the *C. oliveri* in this study. These markers will facilitate the further studies on the mating system, population genetics, and genetic effects of habitat fragmentation of the species. Such information will be useful for developing sound and effective conservation strategies for this endangered species. Furthermore, these developed SSR markers for *C. oliveri* would also be potentially useful for exploring population genetics in the *Cephalotaxus* species.

## Figures and Tables

**Table 1:** Characteristics of 15 polymorphic and 12 monomorphic microsatellite loci for *Cephalotaxus oliveri*.

Locus	Primer sequences(5′-3′)	Repeat motif	Size range (bp)	*T*_a_ (°C)	GenBank accession No.
CO1	F: GTTTACATTGGAGCCAGTCA R: TTTTCCACCTTCTTTGACCT	(AC)**_19_**(AG)**_6_**	173–225	51	JQ902141
CO2	F: GCAACTCATTTGAAGCATCG R: AGGTAGACCAGGCTTCCATT	(GA)**_9_**	235–247	61	JQ902142
CO3	F: TCCTAAATTGAGCCTCTGTG R: TGAAGGTGTTGAGGTGGTCT	(TG)**_26_**	355–359	51	JQ902143
CO4	F: TCAGGCTTTAGATCTTGGAATGT R: GTGGTGGGAGTTCGGAGTTTT	(GA)**_33_**T(AG)**_5_**	257–261	49	JQ902144
CO5	F: CACTGGTGGTTTGCTTGC R: CATCACCTTGCATCCTCA	(TG)**_37_**	170–188	49	JQ902145
CO6	F: CATCTTTTCATAGTTGTCTGC R: CTTAGGTCTAGGTCAAGTGTA	(GT)**_7_**	137–141	55	JQ902146
CO7	F: GAGGAGGTTCAAGGTGGTCT R: CCCACTTCCTCCAGCAATAC	(AC)**_37_**(AG)**_22_**	229–423	66	JQ902147
CO8	F: AATTTATTATGAAGGATAGGG R: CTAATACATTGAATTGGGTAA	(AC)**_35_**	81–137	49	JQ902148
CO9	F: CACAACTCGCAAGAGGTAAA R: GTATCCCTAGTGACTTGGCAT	(CT)**_11_**C**_2_**(CT)**_7_**	174–236	61	JQ902149
CO10	F: AAGAAGAGCAGGTTTGACAT R: GCTACAACTCCCTTGATGAC	A**_10_**	139–143	61	JQ902150
CO11	F: TCATGGGCGCATTAGGAC R: TCTCCTTCGCTCATCAAACTT	(CA)**_45_**	230–338	64	JQ902151
CO12	F: CCAAGTGACCATTCCACCAA R: GTCTCACGTCCATCTTAGCA	(TC)**_7_**	183–191	61	JQ902152
CO13	F: GTGCCCTCATACCCATTCTA R: CCATCACATCTGCCACATCA	(AG)**_9_**	161–173	64	JQ902153
CO14	F: ATCCTGAGTCCCTGTATGTT R: CTACTATCTGAGCACGCCAC	(AG)**_18_**	235–293	55	JQ902154
CO15	F: TCCAAGGATGCACATTCAAT R: AAACAAAACCTCACTCAATGAA	(AC)**_18_**	262–316	58	JQ902155
CO16	F: TTACAAATCAACCACCCC R: GTGCTTGTCAGTGCTAAGGT	(GTG)**_5_**	280	58	JQ902156
CO17	F: ACCTAAGGGTGCTTGGAGATA R: GATGATGACACCTGTTATGCC	(AG)**_6_**	304	61	JQ902157
CO18	F: AGACAATCAACAAGCATACAC R: AACAGGCACATAACTACAAAT	(GA)**_10_**	385	61	JQ902158
CO19	F: AAAACCCAAACAGTGGAAACA R: ACGTTTACGTAGACCTGAATGC	(AC)**_16_**	128	58	JQ902159
CO20	F: TACTAAAACAACTCATTGGAAAC R: TGGTGAAGACCCTGGAAGA	(CT)**_9_**	221	49	JQ902160
CO21	F: ATTGGTGGAAGACCCTAGAAGA R: GCAACTAGGGATGCTAAAACAG	(AG)**_10_**	235	61	JQ902161
CO22	F: AAATATGGAAGAATGGTGATC R: TGATTTAGGGACCTTAGCAC	(AG)**_8_**	150	49	JQ902162
CO23	F: GATGAAGGCACGGGAAAC R: TTGAGAATTGACAAAGTCAAA	(TTTTG)**_3_**	264	49	JQ902163
CO24	F: GGCTTCGGAAGACTACCTT R: AATGACCATGCCAACAAAC	(AG)**_11_**G(GA)**_13_**	165	55	JQ902164
CO25	F: AAAGGCAACATGCTATCAAAT R: TACCGTGTCTTCTGCCTCTGG	(GAA)**_4_**	261	58	JQ902165
CO26	F: AGGGCTATGCTTTGGATGT R: TTGATGAAGTTATGGGTGC	(GAG)**_4_**	288	58	JQ902166
CO27	F: ATTCTTTGTTGGTCCGTTTA R: AGGAGGAGTAGGGATGGTAG	(GAA)**_5_**	175	53	JQ902167

*T*_a_, annealing temperature.

**Table 2:** Genetic characterization of 15 polymorphic microsatellite loci in three natural populations of *Cephalotaxus oliveri.*

		DZ population (*N* = 32)					PB population (*N* = 32)					LX population (*N* = 32)				
				
				HWE					HWE					HWE		
										
Locus	*N*_t_	*N*_a_	*F_n_*	*H*_o_	*H*_e_	*p*-value	*N*_a_	*F_n_*	*H*_o_	*H*_e_	*p*-value	*N*_a_	*F_n_*	*H*_o_	*H*_e_	*p*-value
CO1	22	13	0.139	0.607	0.897	0.0011 *	13	0.080	0.750	0.912	0.0078 ^n.s.^	8	0.004	0.469	0.476	0.5689 ^n.s.^
CO2	6	4	0.249	0.188	0.609	0.0011 *	5	0.280	0.125	0.605	0.0011 *	5	0.177	0.313	0.619	0.0011 *
CO3	2	2	0.120	0.000	0.092	0.0156 ^n.s.^	1	0.001	-	-	-	1	0.001	-	-	-
CO4	3	3	0.000	0.844	0.601	1.0000 ^n.s.^	3	0.000	0.688	0.516	1.0000 ^n.s.^	3	0.000	0.750	0.566	1.0000 ^n.s.^
CO5	5	3	0.000	0.969	0.599	1.0000 ^n.s.^	4	0.000	0.844	0.563	1.0000 ^n.s.^	3	0.000	1.000	0.538	1.0000 ^n.s.^
CO6	3	3	0.000	0.656	0.479	1.0000 ^n.s.^	2	0.000	0.969	0.507	1.0000 ^n.s.^	2	0.000	0.938	0.506	1.0000 ^n.s.^
CO7	33	16	0.000	0.938	0.889	0.9000 ^n.s.^	19	0.059	0.813	0.914	0.0467 ^n.s.^	13	0.073	0.750	0.865	0.0589 ^n.s.^
CO8	22	12	0.068	0.656	0.718	0.1922 ^n.s.^	3	0.085	0.094	0.177	0.0789 ^n.s.^	6	0.000	0.688	0.687	0.6122 ^n.s.^
CO9	26	16	0.000	0.938	0.923	0.7100 ^n.s.^	6	0.000	0.719	0.734	0.5844 ^n.s.^	16	0.000	1.000	0.922	1.0000 ^n.s.^
CO10	3	3	0.000	0.063	0.122	0.0656 ^n.s.^	3	0.000	0.125	0.150	1.0000 ^n.s.^	2	0.000	0.063	0.092	1.0000 ^n.s.^
CO11	25	10	0.000	0.563	0.503	1.0000 ^n.s.^	13	0.000	0.594	0.504	1.0000 ^n.s.^	14	0.000	0.438	0.416	1.0000 ^n.s.^
CO12	5	4	0.002	0.344	0.373	0.5422 ^n.s.^	3	0.000	1.000	0.523	1.0000 ^n.s.^	2	0.000	0.375	0.372	0.8311 ^n.s.^
CO13	6	5	0.096	0.375	0.485	0.0800 ^n.s.^	6	0.183	0.313	0.614	0.0011 *	4	0.000	0.625	0.624	0.6922 ^n.s.^
CO14	19	9	0.219	0.438	0.856	0.0011 *	14	0.018	0.844	0.881	0.3256 ^n.s.^	4	0.000	0.656	0.627	0.7433 ^n.s.^
CO15	26	15	0.205	0.500	0.917	0.0011 *	15	0.000	0.875	0.889	0.4756 ^n.s.^	7	0.000	0.750	0.690	0.9067 ^n.s.^
Mean	13.7	7.9	0.073	0.538	0.604	-	7.3	0.047	0.583	0.566	-	6.0	0.017	0.588	0.533	-

HWE: Hardy-Weinberg equilibrium; *N*_t_: the total number of alleles per locus scored by 96 individuals; *N*_a_: number of alleles per locus and population; *F_n_*: estimated frequency of null alleles per locus; *H*_o_: observed heterozygosity; *H*_e_: expected heterozygosity; *p*-value: *p*-value for exact tests for HWE;

* showed significant deviation from HWE after Bonferroni correction (*p* < 0.00111);

n.s.: not significant.
